# Evaluating the implementation of a hypertension program based on mHealth and community pharmacies integration to primary care centers at a municipality level in Argentina during the COVID-19 pandemic

**DOI:** 10.3389/frhs.2024.1263331

**Published:** 2024-08-08

**Authors:** M. E. Esandi, Z. Ortiz, V. Bernabei, N. B. Villalba, S. Liggio, M. Della Maggiora, N. A. García, A. Bruzzone, G. Blanco, D. Prieto Merino, H. Legido Quigley, P. Perel

**Affiliations:** ^1^ Instituto de Investigaciones Epidemiológicas, Academia Nacional de Medicina de Buenos Aires, Ciudad Autónoma de Buenos Aires, Argentina; ^2^ Departamento de Economía, Universidad Nacional del Sur, Bahía Blanca, Provincia de Buenos Aires, Argentina; ^3^ Secretaría de Salud, Mar del Plata, Municipio de General Pueyrredón, Provincia de Buenos Aires, Argentina; ^4^ Colegio de Farmacéuticos de General Pueyrredón, Mar del Plata, Provincia de Buenos Aires, Argentina; ^5^ IFISUR, Departamento de Física, UNS/CONICET, Bahía Blanca, Provincia de Buenos Aires, Argentina; ^6^ Instituto de Investigaciones Bioquímicas Bahía Blanca, CONICET, Bahía Blanca, Provincia de Buenos Aires, Argentina; ^7^ Argentine Society of Arterial Hypertension, Ciudad Autónoma de Buenos Aires, Argentina; ^8^ Faculty of Medicine, University of Alcalá, Alcalá de Henares, Spain; ^9^ Department of Non-communicable Disease Epidemiology, London School of Hygiene and Tropical Medicine, London, United Kingdom

**Keywords:** hypertension, primary care, community pharmacies, mHealth, implementation research, low- and middle-income countries, pandemic

## Abstract

**Introduction:**

While pharmacists-led interventions in hypertension have proven effective in high-income countries, their implementation and impact in low- and middle-income countries (LMIC) remain limited. This study assessed the implementation and outcomes of the hypertension program FarmaTeCuida (FTC), which integrated community pharmacies into the public primary care level using information and communication technologies. The study took place during the pandemic in General Pueyrredón, Buenos Aires, Argentina, so modifications to the implementation strategy and expected outcomes were also analyzed.

**Methods:**

A mixed-methods study was conducted using the non-adoption, abandonment, scaling-up, dissemination, and sustainability (NASSS) conceptual model. Qualitative in-depth interviews were conducted with key stakeholders using snowball sampling until thematic saturation was achieved. The quantitative approach employed a quasi-experimental, prospective, longitudinal design in a cohort of hypertensive patients enrolled in the FTC program since October 2020 to March 2022. Adoption, access, adherence to follow-up, and blood pressure levels were assessed. Clinical outcomes were compared to a cohort of hypertensive patients attending primary health care centers (PHCCs) in 2021 but not enrolled in the FTC program. Routine data from this cohort was obtained from the municipal health information system (HIS).

**Results:**

Out of 33 PHCCs, 23 adopted the FTC program, but only four collaborated with community pharmacies. A total of 440 patients were recruited, with 399 (91%) enrolled at PHCCs. Hypertension was detected in 63% (279/440) of cases at the first visit (113 were possible hypertensive patients; 26 new hypertensive patients and 140 already diagnosed). During follow-up, FTC identified 52 new hypertensive patients (12% out of 440). Reduction of systolic blood pressure (SBP) was observed in patients enrolled in both the FTC program and the comparison group over 60 days. In the multivariate analysis that included all hypertensive patient (FTC and HIS) we found strong evidence that for each month of follow up, SBP was reduced by 1.12 mmHg; however, we did not find any significant effect of the FTC program on SBP trend (interaction FTC*months has a *p*-value = 0.23). The pandemic was identified as the main reason for the program's underperformance; in addition we identified barriers related to technology, patient suitability, implementation team characteristics, and organizational factors.

**Discussion:**

Our study, grounded in the NASSS model, highlights the profound complexity of introducing innovative strategies in low- and middle-income settings. Despite substantial challenges posed by the pandemic, these obstacles provided valuable insights, identified areas for improvement, and informed strategies essential for reshaping the care paradigm for conditions like hypertension in resource-constrained environments.

## Introduction

Hypertension is one of the main cardiovascular risk factors in Argentina, with a high prevalence of almost 50% in adults aged between 30 and 70. It has an important social, economic, and health impact (1, 2). Nonetheless, despite the availability of highly effective treatments and preventive strategies, only half of hypertensive patients are aware of their condition, and the majority are not adequately controlled (blood pressure < 140/90 mmHg) (3, 4). The flaws in the control of this condition are caused by multiple factors, including health system fragmentation and segmentation, limited population access to health services due to supply and demand issues, particularly in socially vulnerable groups, and failures in the routine, regular, and sustained monitoring of the disease, attributed to both patient and physician factors (5–7).

Different strategies have been shown to improve the timely diagnosis, treatment, and monitoring, resulting in a better control of arterial hypertension (8–10). One of these strategies involves the incorporation of community pharmacies (11).

Clinical trials and meta-analysis have shown the advantages of pharmacist-led interventions (including blood pressure measurement, education, consultation and, even, supervision of medication adequacy) in the improvement of the control of hypertensive patients and in the reduction of hospitalizations rates for cardiovascular events (12–19). Some of the potential factors which explain the effectiveness of this intervention, include: higher attendance of patients to the pharmacy for blood pressure (BP) measurement compared to visiting a general practitioner (20); more frequent and fluid communication with the pharmacist, according to patientś perception (21); proximity (most people have a pharmacy near their residency) (22) and ease of access (no need of scheduled appointment) (23).

Another effective strategy for managing Non-Communicable Diseases (NCDs) is the use of information and communication technologies (ICTs) to facilitate patients-health professionals' interactions. The use of ICTs has specifically demonstrated improved hypertension control in Low and Middle-Income Countries (LMIC), particularly in vulnerable populations (24–26).

In Argentina, the national hypertension guideline from 2019 strongly recommends multidisciplinary team-based care for hypertensive patients, including the involvement of community pharmacists as a strategy to increase treatment adherence and reduce blood pressure (27). Since then, the Argentine Pharmaceutical Confederation has participated in the “*Knowledge and Control Your Blood Pressure*”, promoted by the Argentine Society of Arterial Hypertension, allowing patients to measure their blood pressure for free at registered pharmacies. This campaign primarily aims to increase patient awareness of this condition, but the final diagnosis and treatment depend on the patient's decision to seek medical care at private or public health services. Aside from this initiative, there have been no other initiatives promoting the systematic involvement of community pharmacists in the prevention and control of arterial hypertension and none aimed at integrating their practice with primary health care teams. Although this strategy of care has shown promise in LMICs, there is a lack of evidence regarding its implementation and impact in Latin American countries like Argentina.

As part of the Global Alliance for Chronic Disease Scale Up Program, the National Academy of Buenos Aires, jointly with the Secretary of Health of the Municipality and the College of Pharmacists of General Pueyrredón, and collaborators from the London School of Hygiene & Tropical Medicine, conducted an implementation research study to design, implement and evaluate a control hypertension program, later called “FarmaTeCuida” (FTC), which promoted the integration of community pharmacies into the public primary care level through the use of ICTs. At the time of conducting this research, the municipality lacked information on hypertensive patients receiving care at the primary healthcare level. According to previous studies conducted in the country, it was presumed that a high percentage of these patients were underdiagnosed. Among those aware of their condition, there was a presumption of suboptimal control. Through implementation of the FTC program, we aimed to address this information gap and contribute to reducing the disparities in access and follow-up for these patients by incorporating community pharmacies as an entry point to the system and integrating them into the Primary Health Care Centers (PHCCs). This integration facilitates the referral of hypertensive patients based on the severity of their condition. Originally, the execution of this project was planned for a three-year period, from October 2019 to October 2022. However, the onset of the COVID-19 pandemic in March 2020 forced an adaptation of the program and its implementation strategy, presenting significant challenges for the participating institutions, stakeholders involved in program implementation, and the research team.

The overall aim of this study was to evaluate the implementation and clinical outcomes of this program in the context of a resource-limited setting and how the pandemic context affected its implementation. Specifically, the study objectives were to: (1) evaluate acceptability, adoption, access to the program and adherence to follow-up from the perspectives of patients, implementers, and decision-makers; (2) assess its impact on patient blood pressure levels; (3) assess the value and potential sustainability of the program in the long term as perceived by patients, implementers, and decision-makers; (4) analyze how the irruption of the pandemic affected the implementation of this program and its expected outcomes.

## Material and methods

### Setting

The municipality of General Pueyrredón, Buenos Aires, Argentina, is divided into 31 programmatic areas and has a municipal public health system consisting of 33 PHCCs, which are under the supervision of the Primary Health Care Department of the Secretariat of Health. Each center has a coordinator and a multidisciplinary health team comprised of Family Physicians and/or General Practitioners, nurses, social workers, psychologists, professionals from auxiliary disciplines, and administrative staff. Community pharmacies belong to the private sector and are affiliated with the local College of Pharmacies. The PHCCs located in neighborhoods of high social vulnerability were prioritized by the Secretariat of Health, and nearby pharmacies were invited to participate by the College of Pharmacies.

### Research design

A mixed methods study, consisting in a quasi-experimental, prospective, longitudinal design was conducted.

#### Program participants

Coordinator and health team members working at PHCCs and owners and pharmacists of participating private community pharmacies.

#### Eligible and target population

The eligible population for the study were adult residents of the Municipality of General Pueyrredón (644,055 inhabitants in 2018) who were older than 18 years and gave informed consent to participate. The target population included the following groups: “possible hypertensive patients” (patients with high blood pressure levels in the first visit to pharmacy or PHCC, but that could not be confirmed at PHCCs due to non-return); “new hypertensive patients” (patients with Systolic Blood Pressure > 159 and/or Diastolic Blood Pressure > 100 at the first visit or high blood pressure levels confirmed by a physician at PHCCs in a second visit), and “prevalent hypertensive patients” (already diagnosed or treated hypertensive patients). Pregnant women were excluded.

Two cohorts of hypertensive patients were compared. The first cohort comprised hypertensive patients enrolled in the FTC program from October 2020 to March 2022. The comparison group consisted of hypertensive patients routinely registered in the municipal health information system (HIS) during 2021 but not enrolled in the FTC program.

#### Program implementation procedure

**Figure 1:**
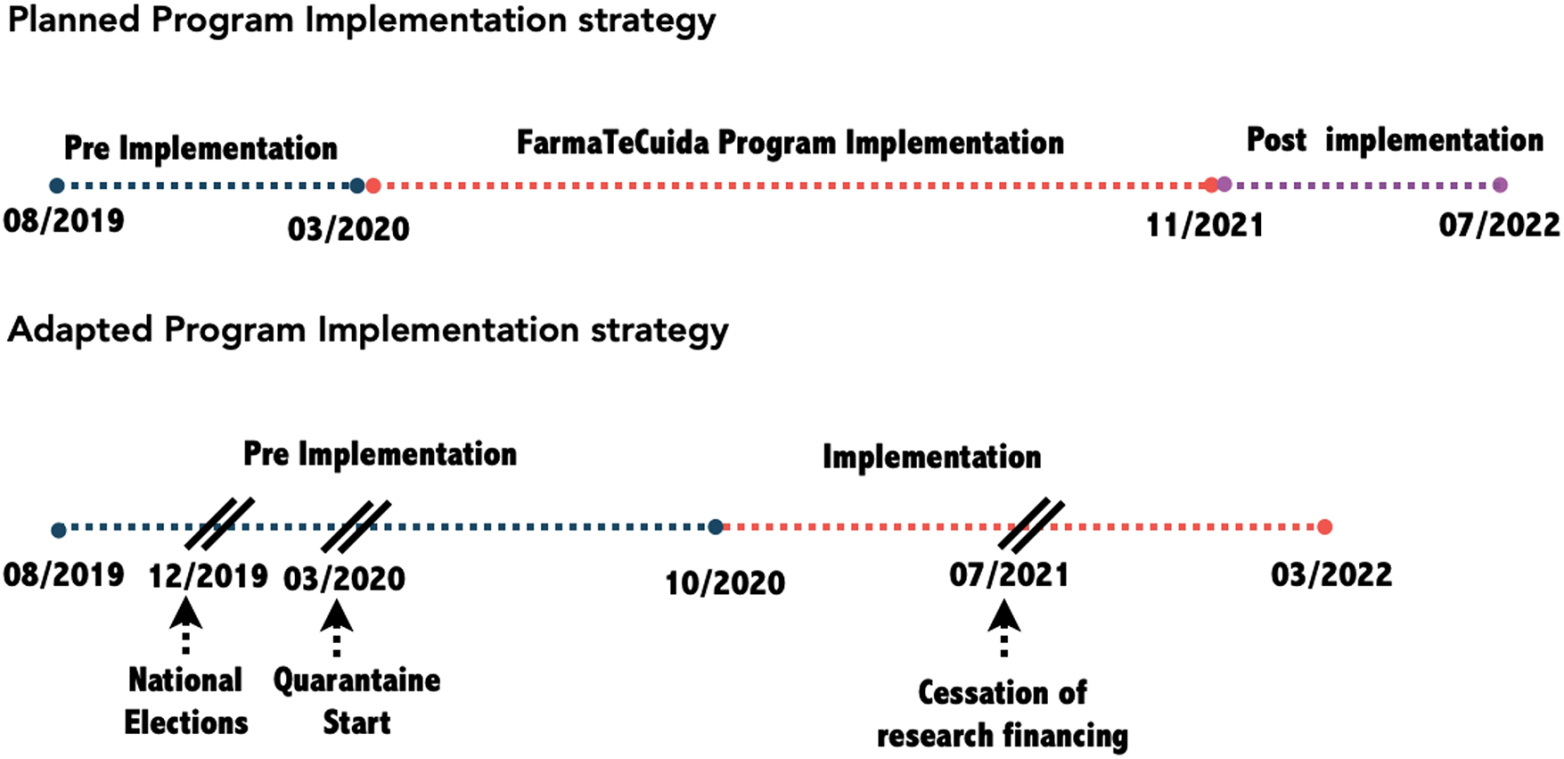
Changes to the programme implementation strategy in the pandemic context.

The core components of the FTC program included the integration of community pharmacies into the public health system through the creation of PHCC-Pharmacy Units, supported by a virtual platform, and the strengthening of patient self-management through the use of a patient-targeted app. Its implementation unfolded in three stages:

*1.* ***Pre-implementation: program adoption*.** It involved the participation of PHCCs and pharmacies, leading to the establishment of Pharmacies-PHCCs Units. PHCCs situated in neighbourhoods with high social vulnerability were given priority by the Secretariat of Health, and neighbouring pharmacies were invited to join the initiative by the College of Pharmacies. The owners and/or pharmacists of each pharmacy were introduced to the coordinator of the PHCC located in the same neighbourhood, facilitating agreements for the creation of the PHCC-Pharmacy Units. Monthly workshops were organized to strengthen this collaboration, including the standardization of referral criteria, diagnosis and treatment practices on the basis of the national hypertension guideline; implementers training through monthly workshops and purchasing and distribution of supplies and equipment to pharmacies and PHCCs. During this stage the virtual platform was developed through a co-design approach, in which implementers (both pharmacists as well as PHCCs coordinators) were actively involved. This platform had different interfaces according to each user profile and was adjusted to implementers requirements during pre-implementation and also once implemented. It allows patient registration and triage on the basis of blood pressure levels and previous diagnosis of hypertension. The patient-targeted app was also developed at this stage. It has an automatized text-messaging system, which was adapted from messages contained in the handbook “*Be Healthy, Be Mobile*”, developed by the World Health Organization and the International Telecommunication Union (ITU) based on a behavior change theory proposed by Michie S (28). Through a participatory approach involving implementers, e-messages were adapted to the local context and an automated e-messages system was incorporated to the app. Antihypertensive drugs were provided to PHCCs according to the Physician's Desk Reference (“*Vademecum*” in Argentina) defined by the Secretariat of Health, which normally guarantees daily delivery of drugs to each PHCC through an established logistics and distribution system. The pre-implementation stage was originally planned from August 2019 to March 2020 but was extended due to the onset of the pandemic.
*2.* ***Implementation: access and adherence by patients*.** It involved the field work, consisting in patient enrolment, diagnosis, treatment and follow-up. The health personnel at participating PHCCs and pharmacies were responsible for identifying, diagnosing, treating, and monitoring hypertensive patients. Patients were recruited either at pharmacies or PHCCs through verification of inclusion criteria informed consent administration, and blood pressure self-monitoring. All enrolled patients were registered on a virtual platform and were informed and instructed to download the patient-targeted app, which contained information on their PHCC and pharmacy visits as well as blood pressure controls. This app also contained an automated text-messaging system that advised patients on healthy lifestyles and appropriate medication use. The diagnosis of newly hypertensive patients was confirmed at PHCCs and treatment decisions were made by the attending physician. Monthly follow-up appointments could be scheduled by patients at PHCCs or participating pharmacies. The role of pharmacists in the hypertension program was to provide counseling on healthy lifestyles and appropriate use of antihypertensive drugs, and to supervise patients' adherence to follow-up and treatment recommendations. They were also involved in recruiting and referring patients to their corresponding PHCC for diagnosis and treatment. Hypertension was defined as Systolic Blood Pressure (SBP) > 139; Diastolic Blood Pressure (DBP) > 89 confirmed in two consecutive visits or a value greater than SBP > 159 and/or DBP > 100 at the first visit; and/or hypertension previously diagnosed and/or under treatment.
  This stage was originally planned to recruit patients during an 18 month-period (March 2020 to December 2021) to allow a 6–12 months follow-up per patient, depending of the enrolment date. Besides, program implementation would be performed progressively, starting in only three PHCCs-Pharmacies Units and scaling-up to the rest of units after all processes and technology tools were piloted, reviewed and adjusted. However, due to the government's imposition of Mandatory Preventive and Compulsory Social Isolation, conducting the pilot test became unfeasible, leading to the postponement of the program's implementation until October 2020—eight months later when the isolation measures were eased. The pandemic disrupted the operational landscape of community pharmacies, resulting in reduced staffing, work reorganization, and constraints on patient entry for blood pressure measurements within the pharmacies. These challenges not only constrained their active involvement but, more significantly, hindered the establishment of PHCC-pharmacy units. Nevertheless, the program's implementation persisted, with a strategic focus on patient recruitment at the PHCCs while also incorporating pharmacies expressing interest in participation.
*3.* ***Post-implementation stage: Procedure of actions for sustainability*.** Originally scheduled for the year 2022, the objective was to enhance program sustainability through active engagement with key stakeholders in deliberative dialogues. However, these dialogues were later substituted with a seminar tailored for the authorities of the participating institutions and the healthcare teams at the PHCCs. The seminar served the purpose of disseminating results and facilitating a reflection on accomplishments that could be sustained under the auspices of the Health Secretariat.
  The three planned stages had to be modified due to the irruption of the COVID-19 pandemic (Figure 1), which, as seen below, had an impact in the whole program implementation. The adaptation process unfolded dynamically, responding to the uncertainties surrounding both the progression of the pandemic and the government's measures to address the crisis. Decisions pertaining to program modifications and strategies for implementation were guided by the project's governance team. This multidisciplinary team included operational representatives from each participating institution and a political component comprising respective authorities. Through a collaborative consensus-building process, the team adeptly navigated the operational and policy decisions essential for advancing the project's implementation.

#### Program evaluation

The overall study was framed with the conceptual model proposed by Greenhalgh T et al., which includes factors that influence not only the adoption of a program or a technology but also its non-adoption, abandonment, scaling-up, dissemination and sustainability (NASSS). This model considers the complexity of innovation implementation at seven different domains including: the condition, the technology or innovation, the value proposition, the adopters, the organizations, the wider system, and embedding and adaptation over time (29).

#### Data collection and outcomes

**Quantitative outcomes:** include program access, adoption and effect on systolic blood pressure (SBP) levels. These data were sourced from records maintained by the healthcare team on the virtual platform for the cohort of patients enrolled in the FTC program, and from the HIS for the comparison group.

*Adoption*: number of enrolled PHCCs and pharmacies; number of centres that effectively recruited patients; number of PHCC-pharmacies units that were created through pharmacists and PHCC coordinators articulation. *Access*: number of recruited patients, numbers of visits. *Adherence to follow-up:* numbers of follow up visits per patient. *Clinical outcomes*: SBP reduction in enrolled patients. A digital blood pressure monitor (Omron, HEM-7121, China) was used both by pharmacists and members of the health team at PHCCs to measure systolic and diastolic blood pressure three times within a 5-minute interval, after a 5-minute resting period in a seated position. Results of each measurement were registered at the FTC virtual platform and the average of these 3 measurements was automatically computed. SBP reduction was measured through secondary analysis of data registered at the FTC virtual platform. In the comparison group, routine data was obtained from the HIS. The implementation of FTC was heavily influenced by COVID (see below) so we focussed our clinical outcome analysis from October 2020 to March 2022 when most of the patient recruitment and follow up took place. The comparison group were all hypertensive patients registered in the HIS during 2021 who were not enrolled in FTC.

**Qualitative outcomes:** The qualitative approach was based on the NASSS conceptual model and provided in-depth insight into the more intangible aspects of the program implementation process, impacts of the new service model on patients, health care workers and other stakeholders and the likelihood of sustainability through its adoption at the institutional level. Barriers and facilitators to program access and adoption and acceptability by users, implementers and decision makers were analysed considering the following NASSS domains: condition, technology, system of adoption, organization and external context. Domains aimed at measuring value of the innovation and its embedding and adaptation over time guide the analysis of the program value attributed by patients, implementers and decision-makers as well as its potential sustainability in the long term.

The collection of qualitative data was conducted through in-depth, face-to-face interviews with key stakeholders to assess the various dimensions of program implementation. Patients, healthcare providers, and other relevant stakeholders including managers and decision-makers were invited to participate in individual interviews. A stakeholder's identification was performed by researchers of participating institutions and other respondents were recruited using snowball sampling techniques until the point of thematic saturation was reached. All selected respondents were asked to sign and date a Spanish-language informed consent form. In-depth interviews were conducted by trained research assistants under the direct supervision of two of the authors (MEE, HLQ). Research assistants were not involved in the implementation of the FTC program. The interviews were recorded and fully transcribed in Spanish.

#### Data analysis

##### Quantitative analysis

We present a descriptive analysis of the patient variables age, sex, education level, SBP and DBP at baseline of the patients recruited in FTC. We stratify this analysis by: (a) location of recruitment (Pharmacy/PHCC), (b) hypertension status at baseline (None/Possible/Newly diagnosed/Prevalent). We then compare these baseline characteristics between patients recruited and not recruited in FTC. We stratified this analysis by whether they had only one or more than one visit in 2021. Finally, we modelled the evolution of SBP levels over follow up visits in 2021 using a linear regression mixed model with random effect by patient to account for the repeated measures. To estimate a time-trend for SBP change we include the number of moths of follow-up in the model (counted from their first FCT visit for those patients enrolled in FTC, or from first visit to the health system in 2021 for those patients that were never enrolled in FCT). To evaluate if this trend was different for patients in FCT we included the binary variable: patient in FTC programme (yes/no) and the interaction between this and the months of follow-up. We also included control variables in the model for age, sex, and month of the year as SBP is well known to show a seasonal cycle.

##### Qualitative analysis

An interpretative approach was used for qualitative analysis. A coding manual was developed based on the domains of the NASSS model. Two reviewers independently conducted the coding and resolved discrepancies through consensus. To enhance the validity and reliability of this analysis, a third reviewer (HLQ) assessed the relevance of the coding with the selected theoretical model. The resulting qualitative data was analysed both deductively (i.e., coding data according to key themes of existing conceptual frameworks) and inductively (i.e., using thematic analysis to elicit new themes or unexpected findings through coding and categorising of data). Analysis was conducted using QSR Nvivo 12 software (license NMP20-JZ000-TH020-AR08Y).

#### Ethical aspects

The study protocol was approved by the Ethics Committee of the National University of Mar del Plata (in compliance with the provincial Law 11.044 on health research) to guarantee that the research envisages the safeguards provided by ethical and legal requirements established by national rules (Law 25326 on Personal Data Protection, ANMAT Provisions 6677/10 and 4008/2017; Ministerial Resolution 1480/11) and international rules (Nuremberg Code, Helsinki Declaration and its amendments; Universal Declaration on the Human Genome and Human Rights approved by the UNESCO General Conference). The study protocol was also approved by the London School of Hygiene & Tropical Medicine Ethics Committee, UK (Reference number: 26418/RR/25089).

## Results

### Quantitative analysis

#### Program adoption by PHCCs and pharmacies

By March 2020, coordinators of twelve PHCCs and owners of twenty-one pharmacies agreed to participate, resulting in the creation of eleven PHCCs-pharmacies units. Three units were chosen to test the operation of the virtual platform and program processes. However, on March 20, 2020, the national government declared the COVID-19 lockdown and all activities were put on hold. Some of the PHCCs were closed and others were prepared and equipped for the pandemic. Even when community pharmacies were not closed, blood pressure monitoring was not permitted as client services were offered outside the building. This took seven months, during which the implementation of the programme could not begin. Virtual sessions were held, primarily to support the interest and engagement of implementers in the program. In October 2020, the eleven units were asked to start patient recruitment, but only two actually initiated patient enrolment at a much lower rate than anticipated. By February 2021, there was a slow uptake of the program, although still lower than expected. In May 2021, the Secretariat of Health hosted a hypertension public campaign to boost patient recruitment at PHCCs. As a result, twenty-three of the thirty-three PHCCs adopted the FTC program. However, many were not associated with a community pharmacy, as their owners refused to continue participating. Only four PHCCs-pharmacy units continued working together until March 2022, when the implementation stage was ended. In the case of the PHCCs that did not have a matched participating pharmacy, recruitment and follow-up was performed exclusively at the PHCCs. In August 2022 and March 2023, meetings were conducted with key stakeholders from the public health and pharmacy sectors to present the main findings ot the study, share lessons learned and discuss potential actions to facilitate future program implementation.

#### Program access by patients and general characteristics of the participants

**Figure 2:**
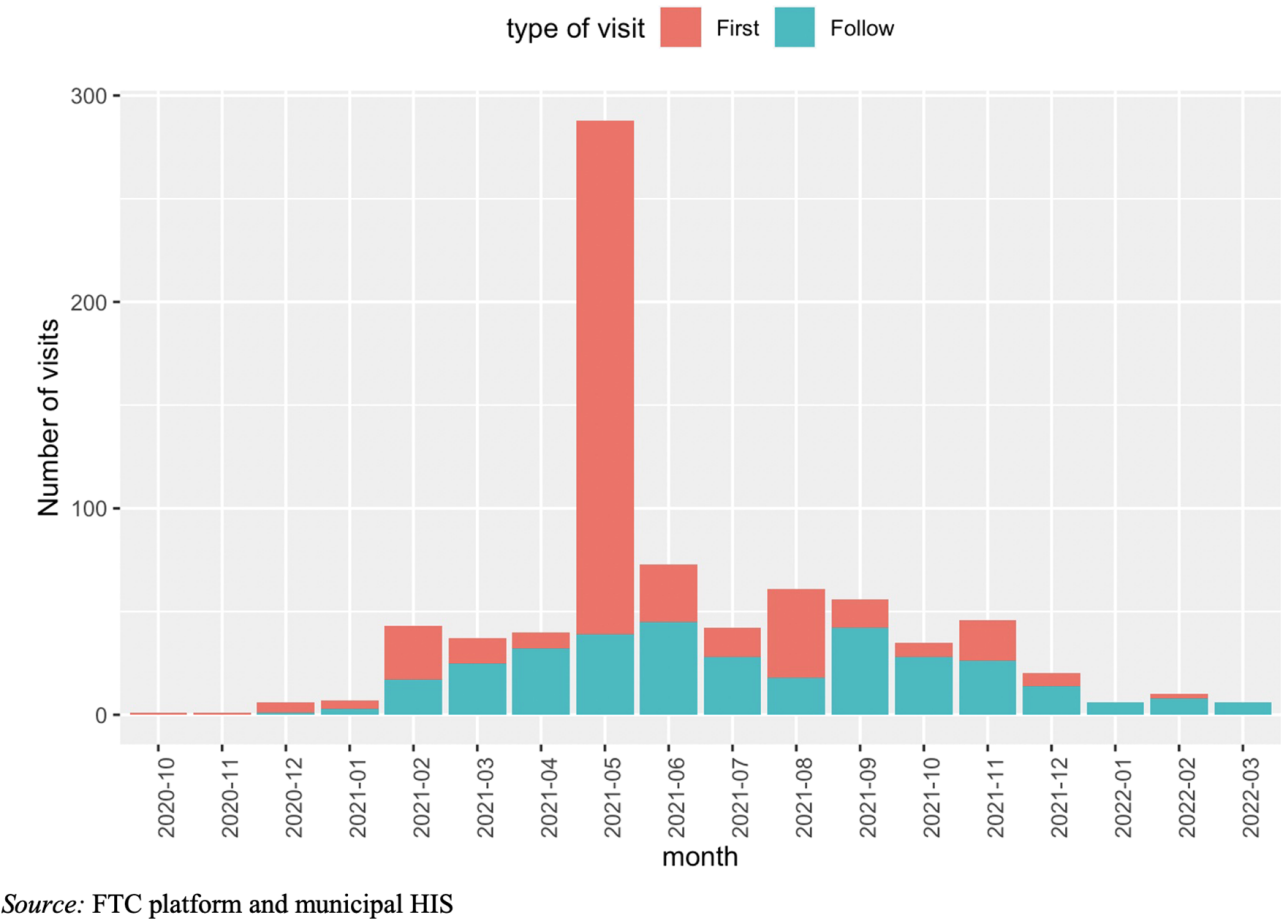
Evolution of first and follow-up visits at the FTC program.

Until March 2022, 440 participants were recruited (Figure 2). Most patients were recruited at PHCCs (399, 91%), being this group slightly younger than the one recruited at pharmacies. Overall mean age (SD) of enrolled patients was 49.3 (24.1) years old, with a higher percentage of women (63%). The education level reached by most patients (73%) was low (incomplete o complete primary level). The mean (SD) SBP at the first FTC visit in the whole population was 138 (21.8) mmHg and the DBP, 86.1 (12.8) mmHg, being higher in the group recruited at pharmacies (Table 1).

**Table 1:** Baseline characteristics stratified by facility at first visit.

	PHCCs (*N* = 399)	Phar (*N* = 41)	Overall (*N* = 440)
Age			
Mean (SD)	52.4 (15.8)	60.0 (16.0)	53.2 (16.0)
Median [min, max]	53.0 [18.3, 86.0]	60.8 [18.7, 92.3]	53.6 [18.3, 92.3]
Missing	29 (7.3%)	1 (2.4%)	30 (6.8%)
Sex			
Female	252 (63.2%)	24 (58.5%)	276 (62.7%)
Male	135 (33.8%)	15 (36.6%)	150 (34.1%)
Missing	12 (3.0%)	2 (4.9%)	14 (3.2%)
Education			
1	38 (9.5%)	6 (14.6%)	44 (10.0%)
2	255 (63.9%)	23 (56.1%)	278 (63.2%)
3	30 (7.5%)	5 (12.2%)	35 (8.0%)
4	18 (4.5%)	6 (14.6%)	24 (5.5%)
5	1 (0.3%)	0 (0%)	1 (0.2%)
6	6 (1.5%)	0 (0%)	6 (1.4%)
Missing	51 (12.8%)	1 (2.4%)	52 (11.8%)
SBP			
Mean (SD)	137 (21.7)	143 (22.7)	138 (21.8)
Median [min, max]	136 [79.0, 210]	142 [90.0, 215]	142 [79.0, 215]
DBP			
Mean (SD)	85.9 (12.9)	87.4 (12.6)	86.1 (12.8)
Median [min, max]	85.3 [48.7, 131]	90.0 [53.0, 113]	85.3 [48.7, 131]

PHCCs, Primary Health Care Centers; Phar, Pharmacies; SBP, systolic blood pressure; DBP, diastolic blood pressure. Source: FTC platform.

At the first visit of FTC, 161 (36%) participants did not have high blood pressure, 113 (25%) were considered as possible cases of hypertension, 26 (6%) were diagnosed as new cases of hypertension, and 140 (32%) as prevalent hypertension. The new hypertensive group had the highest level of SBP and DBP at the first visit (Table 2).

**Table 2:** Baseline characteristics stratified by HT as determined in FIRST visit.

	No HT (*N* = 161)	Possible (*N* = 113)	New (*N* = 26)	Prevalent (*N* = 140)	Overall (*N* = 440)
Age					
Mean (SD)	45.5 (16.7)	56.4 (15.4)	57.7 (13.9)	58.2 (12.5)	53.2 (16.0)
Median	45.0	56.6	54.6	57.6	53.6
[Min, max]	[18.7, 81.8]	[18.3, 85.4]	[29.1, 92.3]	[18.7–91.5]	[18.3, 92.3]
Missing	29 (7.3%)	8 (7.1%)	1 (3.8%)	6 (4.3%)	30 (6.8%)
Sex					
Female	116 (72.0%)	57 (50.4%)	12 (46.2%)	91 (65.0%)	276 (62.7%)
Male	39 (24.2%)	53 (46.9%)	14 (53.8%)	44 (31.4%)	150 (34.1%)
Missing	6 (3.7%)	3 (2.7%)	0 (0%)	5 (3.6%)	14 (3.2%)
Education					
1	7 (4.3%)	4 (3.5%)	6 (23.1%)	27 (19.3%)	44 (10.0%)
2	134 (83.2%)	93 (82.3%)	14 (53.8%)	37 (26.4%)	278 (63.2%)
3	12 (7.5%)	3 (2.7%)	0 (0%)	20 (14.3%)	35 (8.0%)
4	4 (2.5%)	2 (1.8%)	3 (11.5%)	15 (10.7%)	24 (5.5%)
5	0 (0%)	0 (0%)	1 (3.8%)	0 (0%)	1 (0.2%
6	4 (2.5%)	1 (0.9%)	0 (0%)	1 (0.7%)	6 (1.4%)
Missing	0 (0%)	10 (8.8%)	2 (7.7%)	40 (28.6%)	52 (11.8%)
SBP					
Mean (SD)	119 (11.5)	148 (14.7)	169 (24.5)	146 (17.8)	138 (21.8)
Median	119	147	168	144	137
[Min, max]	[79.0, 139]	[90.0, 179]	[123,215]	[111,195]	[79.0, 215]
DBP					
Mean (SD)	76.1 (8.61)	92.9 (8.51)	104 (13.3)	88.8 (11.2)	86.1 (12.8)
Median	78.0	92.7	102	86.7	85.3
[Min, max]	[48.7, 89.7]	[60.7, 110]	[81.7,131]	[62.0, 126]	[48.7, 131]

Source: FTC platform and municipal HIS. SBP, systolic blood pressure; DBP, diastolic blood pressure.

1: None or incomplete primary education.

2: Complete primary education.

3: Incomplete secondary education.

4: Complete secondary education.

5: Incomplete tertiary or university education.

6: Complete tertiary or university education.

A total of 3,168 hypertensive patients were registered at the HIS from January to December 2021 and were included in the comparison group. Among this group, 1,371 (43%) had only one visit and 1,797 (57%) more than one. The patients that had only one visit in FCT were significantly younger, with lower SBP and DBP and a higher proportion of women compared to those with only one visit in HIS. However, patients in FCT and HIS with more than one visit were not significantly different at baseline in age, gender and SBP levels (Table 3).

**Table 3:** Baseline characteristics depending on enrollment in FTC and number of visits.

	Only 1 visit, *N* = 1,710			More than 1 visit, *N* = 1,898		
	FTC, *N* = 339^a^	HIS, *N* = 1371^a^	*p*-value^b^	FTC, *N* = 101^a^	HIS, *N* = 1797^a^	*p*-value^b^
Age	52 (17)	55 (13)	<0.001	57 (13)	54 (13)	0.046
Unknown	26	0		4	0	
Sex						
Female	219 (67%)	772 (57%)		57 (59%)	1.117 (62%)	
Male	110 (33%)	590 (43%)		40 (41%)	677 (38%)	
Unknown	10	9		4	3	
SBP	135 (20)	149 (24)	<0.001	149 (23)	147 (23)	0.4
DBP	84 (12)	90 (14)	<0.001	93 (13)	88 (13)	0.002

^a^ Mean (SD); *n* (%).

^b^ Welch Two Sample *t*-test; Pearsońs Chi-squared test.

Source: FTC platform and municipal HIS.

#### Follow-up and program effect on blood pressure

Most of enrolled patients (77%, 339) never returned and had just one visit. Of the 101 who returned final diagnoses were as follow: 10 were not considered as hypertensive, 1 as possible, 39 as new and 51 as prevalent cases.

**Figure 3:**
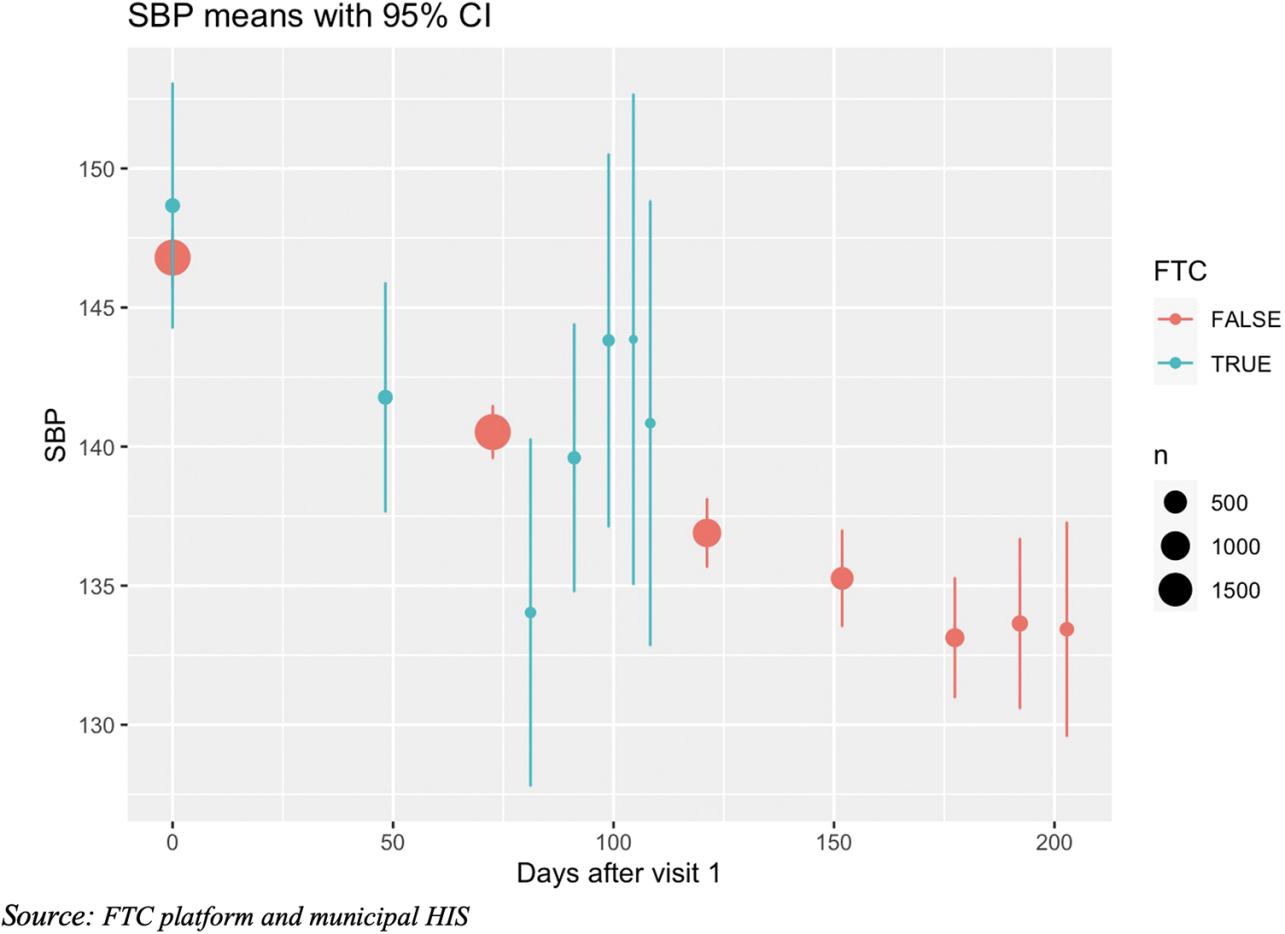
Evolution of SBP over visits by HT condition (mean and CI95%).

Figure 3 shows the evolution in SBP pressure across different visits (the x-axis is the average number of days to the follow-up visit from visit 1) for both patients enrolled both at the FTC program and the comparison group from HIS. It can be seen that a reduction in SBP was observed in both groups (although the small sample size in the FTC group makes the estimates very uncertain).

In the multivariable analysis (Table 4) that included all hypertensive patient (FTC and HIS) we found strong evidence that for each month of follow up SBP was reduced by 1.12 mmHg, we also found strong evidence that age and being male were associated with higher SBP and that SBP was lower in the summer months (January to March), however we did not find any significant effect of the FTC program on SBP trend (interaction FTC*months has a *p*-value = 0.23).

**Table 4:** Mixed effects linear model of SBP mean over follow-up.

	sbp		
Predictors	Estimates	CI	*p*
First visit	**138**.**24**	**136.37–140.11**	**<0**.**001**
If in FTC	−0.20	−3.60–3.21	0.909
Per month of follow-up	**−1**.**12**	**−1.32** to **−0.91**	**<0**.**001**
Per year of age	**0**.**16**	**0.10–0.21**	**<0**.**001**
Male—Female	**4**.**60**	**3.12–6.08**	**<0**.**001**
Month: 2	0.84	−1.88–3.56	0.546
Month: 3	1.79	−0.68–4.27	0.156
Month: 4	2.24	−0.26–4.74	0.079
Month: 5	**4**.**74**	**2.27–7.20**	**<0**.**001**
Month: 6	**6**.**85**	**4.58–9.11**	**<0**.**001**
Month: 7	**6**.**54**	**4.27–8.81**	**<0**.**001**
Month: 8	**6**.**68**	**4.46–8.90**	**<0**.**001**
Month: 9	**6**.**67**	**4.49–8.85**	**<0**.**001**
Month: 10	**3**.**66**	**1.45–5.86**	**0**.**001**
Month: 11	**3**.**74**	**1.48–5.99**	**0**.**001**
Month: 12	**3**.**48**	**1.07–5.89**	**0**.**005**
Interaction FTC*months	0.40	−0.23–1.03	0.211
Random Effects			
*σ* ^2^	289.17		
*ι*_oodni_	147.08		
ICC	0.34		
N_dni_	1892		
Observations	6089		
Marginal *R*^2^/Conditional *R*^2^	0.058/0.376		

Source: FTC platform and municipal HIS.

Bold values represent that results are statistically significant.

#### Qualitative analysis

We conducted interviews with 32 individuals, including decision-makers, implementers, users and also, researchers. Their caracteristics are shown in Table 5. We present findings on barriers and facilitators, value and sustainability of the program in the long term according to NASSS domains.

**Table 5:** Characteristics of interviewee individuals.

	Female	Male	Total
Age range (years)			
25–45	4	4	**8**
46–59	18	4	**22**
More than 59	0	2	**2**
Institution			
Secretary of Health (SoH)	6	0	**6**
Collegue of Pharmacists (CoPh)	2	1	**3**
Primary Health Care Centers (PHCC)	12	2	**14**
Pharmacies (Ph)	2	5	**7**
Research Institution	1	1	**2**
Role			
Decisor (SoH)	4	0	**4**
Decisor (CoPh)	1	1	**2**
Implementer (PHCC & Ph)	12	8	**20**
Patient	4	0	**4**
Researcher	1	1	**2**

Bold values represent that results are statistically significant.

#### Barriers and facilitators to access and adoption of the FTC program

##### Condition: target population characteristics

Many respondents, including patients themselves, reported that users in general had little awareness of their own health, little understanding of the health system, and limited digital literacy. This lack of knowledge hindered program access and adoption, specially among older adults. For example, one user stated: “*Why do you think it might not have worked? Perhaps due to lack of connectivity of people. It could be. I think the problem with that type of apps is that older people may not have much experience with the internet or connectivity.”* (Case 17, user). An implementer noted the lack of access to technology among seniors as a barrier, saying: “*Grandparents do not have technological means.. Many people don't have email, they don't know what an email is. That's a barrier*” (Case 27, implementer, pharmacy). The FTC program aims to empower hypertensive patients in self-care, but several interviewees found it challenging to modify behavior patterns related to preventive controls, treatment, and follow-up at PHCCs due to not taking hypertension serioulsy as a condition. One user said: “*I didn't come to the doctor or do the controls until I felt bad” (Case 15, user*). When inquiring about the useŕs adherence to the program, some interviewees pointed out differences depending on the individual socio-economic situation: “*It may work for a certain sector. Not for everyone,”* referred one of the implementers, reinforcing the need for a greater adjustment between the program and the characteristics of the target population (Table 6). These barriers highlight the need for better alignment between the program and target population.

**Table 6:** Barriers and facilitators according to NASSS domains from the perspective of multiple stakeholders.

NASSS domain	Impact on FTC adoption	Interviewee	Expression
Condition	Barrier related to patient condition	Case 12, Implementer PHCC	*“How do you see the use of technology in this context you work in (referring to the app for patients)? It may work for a certain sector, but not for everyone. Imagine a population that doesn't even have internet. I don't see it as very feasible. If they have access to a phone and a network, it's feasible. There are people who don't have it. It would work for some, and not for others.”*
Technology/Adoption System	Characteristics of the technology as a barrier	Case 3 decision maker, Secretariat of Health	*“And the Program's platform, were you able to see it? Yes… it seemed kind of difficult, right? Somewhat slow for the pace of life and the variety of activities one engages in, don't you think? Having to register information and not having agility in doing so seems to me that it should be more basic… or more… "agile” is the word, right?"*
Technology/Adoption System	Characteristics of the technology as a barrier to program adoption	Case 19, implementer, pharmacy	*“At some point, we wanted to upload [referring to patients ‘data] and couldn't. It didn't seem agile to me. I've been told, and I tried it once and it was a bit difficult for me. I got upset and that was it."*
Organization/Adoption System	Working conditions as a barrier to program adoption	Case 28, implementer, pharmacy Case 11, implementer PHCC	*"I'm very motivated and this program is very good, but it's hard to implement when you have few staff and a lot of work.”* *“When we didn't have the computer, some were left hanging. Other times we had cable theft. I repeat, here it's very desolate, they steal your cables to sell them, etc.etc. So we don't have connectivity to be able to connect."”.*
Adoption System	Individual characteristics as a barrier	Case 8, implementer pharmacy	*"At some point, one of the doctors (at the PHCC) told me they were not interested in the program. I kept that in mind. There you have one less recruiter. The involvement of the doctors is important. That can be a weak point.*
Adoption System	Individual characteristics as a facilitator	Case 10, implementer, PHCC	*"Do you think that adopting this type of program depends on the place or the person? It depends on the person and if they're dedicated*. *And what characteristics does the professional have? Commitment to follow through, taking care of the work. And being perseverant. Yes, that's essential because sometimes you're tired or have certain difficulties in the middle, like we do."*
Organization	Lack of resources as a barrier	Case 12, Implementer, PHCC	*"We have a large population, and the programmatic area has grown a lot, and the clinic infrastructure is too small. We ran out of office space, imagine we're talking inside a kitchen, which we converted into an office. And there are more specialties too. What we need is at least two more offices, and more doctors as well, because two general practitioners are not enough. But we can't have more doctors because we only have two offices. We don't have physical space."*
Organization	Articulation with other municipal programs as a facilitator	Case 21, decision maker, Secretariat of Health	*In May, there was a campaign for World Hypertension Day, but we did it throughout the month of May, from May 10th I think, until June 10th. We took 30 days and did it all together. Farmatecuida and the hypertension campaign went from PHCC to PHCC doing blood pressure checks and identifying patients in order to merge the two databases."*
External Context	Pandemic as a barrier	*Case 27, Implementer, Pharmacy* *Case 15, patient*	*…But then the pandemic hit. And then we tried to organize ourselves, we had several meetings, then we had several Zoom meetings. I think I couldn't attend the last few meetings because during working hours, in the middle of the pandemic, it was impossible for me to connect.…”* *“It was horrible to come here to get checked because you had to wait outside, they had to announce you, disinfect you, until you were allowed in and attended to. We couldn't have more than one or two people inside, so we were all waiting outside. It was a bit complicated."*

Among facilitators, interviewees from community pharmacies identified that patients prefer to have their blood pressure checked at the pharmacy, especially in low-income neighborhoods. One implementer stated, “*It's more comfortable for patients to check their blood pressure at the pharmacy. Vulnerable patients also tend to go to pharmacies in low-income areas”* (Case 9, implementer, pharmacy).

##### Technology: the FTC platform

Lack of fit between technology and user characteristics, including digital illiteracy and limited access to resources such as internet and connectivity, was identified as a major barrier to program adoption by a high number of users and implementers. An implementer noted, “*And what about the patient and technology? I saw the patient doing well, as if he were interested. Perhaps technology is a gap that we have, especially since not everyone has access to the internet. However, most people do have a phone. I believe this is a significant obstacle.”* (Case 23, implementer, Secretary of Health). Some interviewees highlighted the benefits of the information generated by the platform, but many reported concerns related to data collection. These included large amount of data required, long loading time, incompatibility with other health agent tasks and serious constraints in using the platform simultaneously with patient assistance. One implementer stated, “*I couldn't keep up with recording everything the program asked for while I had internet, which would be ideal. I tried but it took too much time, so I chose to do it at the end of the day. Although it was important, doing it in real-time was complicated*.” (Case 10, implementer, PHCC). Integrating the FTC virtual platform with the HIS was seen by some respondents as crucial for the program's adoption and sustainability. It was reported that duplication creates major difficulties for the health team at the PHCC, as it requires double data entry for each patient in different information systems. The coordinator of one of the PHCCs noted, “*Are the data duplicated in the HIS? Yes, because they are also entered into the HIS.*” (Case 10, PHCC implementer).

Repeated attempts from the research team to achieve this integration during program implementation were unsuccessful despite its technical feasibility. A programmer from the research team noted, “*To overcome this issue and enhance both systems, we considered creating interoperability between them, which is technically possible*.” (Case 30, IT research team).

##### Adoption system: decision makers, PHCC teams, and pharmacists

Several interviewees reported that the relationship between pharmacies and PHCCs was complex and varied greatly across different units. A pharmacist stated, “*I found the project interesting, but what I was most looking forward to was the connection with the primary healthcare center, which has proven to be difficult.”* (Case 27, implementer, pharmacy). The coordinator of the PHCC belonging to the same unit said: “*The FTC enabled me to get to know pharmacists and collaborate with them. However, the pandemic interrupted our interactions.” (Case 26, Implementer, PHCC)*.

The involvement of PHCCs teams and community pharmacy staff was viewed positively by some interviewees at the beginning of the project. However, they also reported a drop in their participation and commitment, with the pandemic and quarantine being reported as initial causes. One interviewee stated, “*Do you think the FTC would have worked well if it weren't for the pandemic? I think we had everything in place. Maybe there would have been other issues because of the change in management, but we had everything set up—designated pharmacies, pharmacists working—it just needed to be continued. It seems to me that it would have worked.*” (Case 5, decision maker, Pharmacy).

Beyond the pandemic's impact and the technology problems previously mentioned, other factors hindered FTC adoption, according to several interviewees. Working conditions, such as heavy workloads, time restriction, inadequate staffing as well as leadership and team collaboration problems strongly affected the adoption of the program. These problems were worsened by external factors, including the pandemic. Several interviewees highlighted that adoption and participation in the program by members of the health team strongly depends on individualś characteristics, like interests, commitment and perseverance (Table 6).

##### Organization: the municipal health system and community pharmacies

In addition to the pandemic, structural factors of the local health system, including insufficient resources, limited access to primary care appointments and system fragmentation hindered the adoption of the FTC program. These issues made it challenging for patients to be referred and counter-referred between different levels of care. Many respondents reported a shortage of health personnel (due to recruitment difficulties and lack of incentives, particularly financial) and of critical supplies for program operation, such as medication and other resources (e.g., internet connectivity, phones, computers, and blood pressure monitors). The limitations of the physical environment in some PHCCs, including insufficient consulting rooms and pharmacy space, were also mentioned as factors that hindered the implementation of the FTC program (Table 6). One respondent stated, “*Some of the main PHCCs have been without phones for months due to cable theft.”* (Case 22, decision maker).

Many respondents emphasized the importance of considering health teams' soft skills when adopting this type of innovations, such as leadership, teamwork, willingness to innovate, commitment, and empathy. Some interviewees emphasized the importance of building trust with the community for successful implementation of this type of initiative. One implementer at a pharmacy said, “*Many people consult me first and then go to the doctor. It's almost classic.*” (Case 27) Another highlighted the heterogeneity of the PHCCs in terms of these soft skills: “*PHCCs aren't all the same, it depends on leadership, training, and professional communication. The coordinator's profession also matters.*” (Case 23, Implementer, Health Secretary).

Respondents noted that other municipal initiatives would facilitate implementation of some of the components of the FTC program, such as the “*Healthy Corners*” program (mobile units for health checks), PROTEGER (a national program aimed at preventing chronic diseases including hypertension) and a hypertension campaign annually organized by the Argentinian Society of Arterial Hypertension and the municipality. Objectives of these initiatives are aligned with those of FTC and can contribute to its successful implementation in the long term.

##### Perceived value attributed by users, implementers and decision-makers

Most interviewees agreed on the value of the program and the use of ICTs for the user, health team, pharmacies, decision-makers, and research team. Some decision-makers and implementers highlighted the program's strengths, such as providing management information, improving blood pressure control and patient health, and benefits for participating pharmacies:

*"What helps you is to get results faster, better indicators in PROTEGER. That is, the fact that FTC functions perfectly, allows us to quickly get a better compliance with indicators (…) and better compliance with indicators means more resources."(Case 21, Decision maker, Health Secretariat)*

*"Well and in your experience, what do you think the program has brought you? I liked it a lot that they made us participate because the health center is located in a remote area of Mar del Plata, as it is a very special population and we are few people. But the fact of having been summoned to us enthused me, it enthused me a lot. And the observation that was made towards us, the importance they gave us as a health center. That brought us a lot of growth. Also being able to order ourselves with the patients in this case hypertensive. (Case 10, Implementer, PHCC Coordinator)*.

*"The pharmacy wins that, a loyalty to patient adherence. What people look for is that. Good attention, good service. “(Case 20, implementer, Pharmacy).”*

This initiative was also valued by researchers. One saw the opportunity to participate in the project as a way to contribute to the improvement of the health system, and to redefine the role of research as a means to achieve that goal: “*I think that researching while doing the task within the systems is fundamental, it's crucial. And I'm very happy that we have arrived at the end because we are better than when we started*.” (Case 31, researcher)

##### External context: professional and regulatory framework

According to some of the interviewees, the regulatory framework and current pharmacy model of care negatively affected the implementation of the integrated strategy proposed by the FTC program. The current pharmacy model is predominately based on profitability and the sale of medications. The provision of certain practices, such as blood pressure monitoring, is not legally permitted. Despite being unauthorized, many pharmacies offer blood pressure monitoring for a fee, which creates a barrier to the program's sustainability, particularly for vulnerable people who cannot pay. During the research, self-monitoring was provided free of charge. In relation to this point, one of the interviewees stated: “*One concern I had was that pharmacies are not authorized to control blood pressure in the province of Buenos Aires, but nevertheless, they all do it. I'm talking to you legally, normatively. Participation of the former director of regulation in the project had the sense of incorporating this practice into the system.*” (Case 31, researcher). Regarding the impact of the legal framework on adoption of the FTC program one of the interviewees expressed: “*The law mandates that the pharmacist must own the pharmacy, but some pharmacies have a pharmacist who only owns 1% of the shares. These pharmacies operate with a precarious society structure called a simple limited partnership, where the pharmacist is a limited partner with 1%–2% and the non-professional general partner holds 98% of the shares. In reality, this partner makes all management decisions.”* (Case 1, decision maker).

##### The impact of the pandemic

The majority of participants pointed out that the pandemic was the main reason the project did not work as it was expected. This was due to its impact on two of the project's pillars—the PHCCs and pharmacies—as well as on the population, who stopped getting checked. The interviewees agreed that maintaining involvement in the program during the pandemic was challenging, despite efforts to keep it going:

*"Bringing these two sectors together to do this work and in a pandemic… was like crossing the Sahara in a storm… (Case 4, decision maker)*

*"First and foremost, because of the context we faced, which made everything difficult…I insist, it was a bad time to implement it. I'm sure that in another context the project would have had different results, I'm sure. But well, the context killed us” (Case 25, implementer, pharmacy)*.

##### Sustainability and potential of the FTC program in the long term

Several of the interviewees highlighted the need to link the FTC program with other municipal programs, such as PROTEGER, and integrate its platform into the HIS in order to strengthen its implementation in the future: “*…that FTC can be unified to the HIS and PROTEGER in everything related to information and communication technologies. What for? To avoid double work…Double or triple work. Because getting data for the PROTEGER is also extremely difficult…” (Case 22, decision-maker).* Several participants saw potential for the FTC program to be spread for other illnesses, like diabetes, and scaled-up to other municipalities and sectors of the health system, including the private sector. A few interviewees from the pharmacy sector emphasized the potential of the program to facilitate a paradigm change in the model of care provided by community pharmacies: “*Pharmacies in the future have to provide a service, they can't just sell medicines, they have to be a service. Among these services, we can include both blood pressure monitoring and the screening for many other diseases*” (Case 20, implementer, pharmacy).

## Discussion

The findings of this study highlight the extreme complexity of implementing an intervention like the FTC program in a resource-constrained context, especially during a pandemic. The complexities of the intervention, the health system, and the external context had a multiplier effect that made it extremely difficult to manage. The program's adoption was limited to PHCCs, with pharmacies' participation being practically non-existent, as only a few accepted to participate and coordinate with their corresponding PHCCs. Even though initial institutional arrangements between the public and private sectors were established, and enthusiasm and commitment were high, the pandemic had a profound impact at the organizational and individual level. As expected, the program showed no effect on controlling hypertension among enrolled patients. The qualitative analysis showed that although the pandemic primarily explained the non-adoption of the program, other factors, such as the misalignment between the technology and the target population, structural factors within the health system, and soft skills of the health team (such as leadership, communication, commitment to the task, and teamwork), strongly influenced program implementation.

It is well knwon that implementation of interventions designed to improve clinical and/or health outcomes often proceeds differently from what is planned. Moreover, unexpected external challenges might appear and it is critical to review them in order to learn and share lessons of these unanticipated challenges. In this study, aimed to evaluate the implementation of a hypertension program based on mHealth and community pharmacies integration to PHCCs at a municipality level, we faced both unexpected major events such as the COVID-19 pandemic and expected challenges translated in opportunities and lessons learned that can help future implementation efforts.

For a better understanding we grouped the implications of our findings and lessons learned into four categories: (i) the NASSS model, (ii) the complex interventions, (iii) the implementation research process; and (iv) conducting implementation research in LMIC.

(i) Utilization of a theoretical model to guide implementation efforts is critical to overcoming challenges and to facilitating successful uptake of an intervention. The NASSS conceptual model is aimed at identifying the different areas of complexity (uncertainties, interdependencies and possible unintended outcomes) that emerge when implementing a technology project. It address factors that influence not only the adoption of a program or a technology but also its non-adoption, abandonment, scaling-up, dissemination and sustainability and provide guidance to simplify complex aspects and/or mitigate risks in order to increase the program feasibility and probability of success. The use of the NASSS model helped us to recognize the multiple and imbricated factors that limited the adoption of the program, even when it was highly valued by the majority of stakeholders. Simplifying and strengthening the fit of the FTC program's virtual platform to its targeted users, formally articulating it with other municipal programs, integrating it into the municipal HIS, and considering staff soft skills when prioritizing PHCCs and pharmacies for implementation are essential elements to consider for promoting the program's adoption and ensuring its sustainability in the medium and long term.
(ii) Complexity in this research arose not only from the intervention itself, which involved prevention, diagnosis, and treatment components, but also from multiple stakeholders, multiple outcomes, and a system change that involved integrating community pharmacies into the public health system. The implementation of the program was also complex and was exacerbated by the outbreak of the pandemic, which altered government priorities, temporarily closed PHCCs, and required the modification of care protocols at pharmacies. The unexpected crisis in the health system had a negative effect on the implementation of the program, which had to be adjusted to maintain the project. However, the biggest learning was that in order to maintain the program, it was crucial to redirect efforts without neglecting contact and work with teams. If we, as researchers, had reasoned that it was not possible to research the implementation of the hypertension program due to the pandemic and had discontinued the project, we would not have learned that crisis, emergency, or circumstances should not deviate us from our research objectives when using this methodology, designed to evaluate real-life situations that generate real-world evidence, and whose systematization is key to contribute, for example, as the care for people with chronic diseases was greatly impacted by the pandemic.
(iii) The implementation research process gave us many learning opportunities beyond the pandemic's impact on the intervention. The implementation of hypertension prevention and control programs based on innovative care strategies that have been successful in developed countries presents enormous challenges in resource-constrained contexts. Although the program was implemented, its low adoption and problems related to implementation quality strongly limited the possibility of assessing the effect of pharmacist-led interventions as it was demonstrated in recent meta-analyses (12–19). Nevertheless, the research had significant learnings from the public health, health services, and research fields, as it was the first implementation research in Argentina in which a hypertension program based on this innovative care strategy was designed and implemented involving multiple actors in the health system. Adoption, maintainance and sustainability of this kind of program in the long term implies a paradigm shift in the care of patients with non-communicable diseases, therefore it will require years to occur. This study played a crucial role in sparking the discussion about the role of community pharmacies in the healthcare system, promoting the creation of new partnerships among stakeholders and connecting them through technology. Evaluation activities involved stakeholders from different sectors and organizations throughout the research. Communication of its results to health authorities and members of the health team contributed to identifying opportunities for improvement in many organizational processes that the FTC program implementation depended on, such as hypertensive medication acquisition and distribution, PHCCs structure, and internet connectivity, among others. Results of qualitative assessment also emphasized the impact of soft skills on the acceptance or rejection of new ideas, particularly in settings that are dominated by informal processes, which increase the importance of individuals' motivation and capabilities as drivers of change. Finally, this experience showed the need to integrate new technologies to local information systems and embeb programs like FTC into existing municipal related programs to favor its adoption.
(iv) The implementation of the study during the pandemic exacerbated the impact of multiple barriers that prevails in resource constrained settings and were identified with qualitative assessment, but most of them are structural and will continue to affect the implementation of this type of program in a post-pandemic context. In health systems with scarce and limited resources, future research for the evaluation of such complex innovations requires the consideration of strategies that allow for the generation and strengthening of these resources and the necessary preparation of the health system to enable adoption. This probably double or triple the time that is required to implementing innovations like the FTC program in comparison to high income countries, in which resources is not a critical issue but just another contextual element to consider in the conceptual models or theoretical frameworks on which the research is based. In LMIC, resources become the study object, as it was mentioned by Yapa and Bärnighausen (30).

The research had significant limitations in interpreting results related to clinical outcomes, primarily due to challenges in assessing the impact of pharmacy participation on hypertension control, given the limited number of pharmacies that adopted the program. This study is strengthened by its utilization of real-world data from the Municipality's health information system, facilitating a comprehensive analysis of all patients diagnosed with hypertension in primary care—a feat previously hindered by resource constraints. However, a limitation stems from the quasi-experimental design mandated by the nature of real-world data. While the study successfully compared age and sex distributions, other variables remained unassessed due to inherent limitations in the information source. As randomized patient allocation was not feasible, multivariate analysis was conducted to adjust for potential confounders. Despite these constraints, we believe that the insights gained from the entire implementation process could be valuable for other low- and middle-income settings.

## Conclusion

This implementation research based on the NASSS conceptual model evidenced the huge complexity involved in the implementation of this type of innovative strategies in the context of LMIC. The pandemic undoubtedly greatly hindered the execution of the program, but it was precisely these obstacles that allowed to learn, identify improvement opportunities and design the strategies that are required to favor changes in the paradigm of care for diseases such as hypertension in these settings. It is necessary to look beyond the obstacles and identify the multiple structural factors that affect the adoption of innovations in resource-constrained settings, because these factors continue to be present and many have worsened after the pandemic. Implementation research in these contexts is complex, frustrating, challenging, but absolutely necessary to drive the changes that the system needs to respond to the health needs of the population.

## Data Availability

The datasets presented in this article are not readily available because dataset are owned by the municipal public health system. Requests to access the datasets should be directed to eesandi@gmail.com.
